# Long-term disease-specific and cognitive quality of life after intensity-modulated radiation therapy: a cross-sectional survey of nasopharyngeal carcinoma survivors

**DOI:** 10.1186/s13014-016-0704-9

**Published:** 2016-09-26

**Authors:** Alan Kiang, Vivian K. Weinberg, Ka Ho Nicholas Cheung, Erin Shugard, Josephine Chen, Jeanne M. Quivey, Sue S. Yom

**Affiliations:** 1 Department of Radiation Oncology, University of California, San Francisco, USA; 2 Helen Diller Family Comprehensive Cancer Center, 1600 Divisadero St, MZ Bldg R H1031, Box 1708, San Francisco, CA 94143-1708 USA

**Keywords:** General quality of life, Cognitive quality of life, Intensity-modulated radiation therapy (IMRT), Nasopharyngeal carcinoma (NPC), Functional assessment of cancer therapy (FACT)

## Abstract

**Background:**

There is a lack of data on quality of life in long-term survivors of nasopharyngeal carcinoma (NPC) who have been treated with intensity-modulated radiation therapy (IMRT). We characterized long-term disease-specific and cognitive QoL in NPC survivors after IMRT.

**Methods:**

We conducted a cross-sectional study of surviving patients diagnosed and treated for NPC at our center with curative-intent IMRT, with or without chemotherapy. Patients who were deceased, still undergoing treatment, with known recurrent disease, or treated with RT modality other than IMRT were excluded. QoL was measured by FACT-NP and FACT-Cog.

**Results:**

Between May and November 2013, 44 patients completed cognitive (FACT-Cog), general (FACT-G), and NPC-specific (NPCS) QoL assessments. Patients were categorized into 4 cohorts based on duration since IMRT (≤2.5, >2.5–6, >6–10, and >10–16 years). There was no significant difference in age (*p* = 0.20) or stage ((I/II vs III/IV: *p* = 0.78) among the cohorts. The 4 cohorts differed overall for all QoL measures (ANOVA: *p* < 0.02 for each), due to improved scores >2.5–6 years post-IMRT compared with ≤2.5 years post-IMRT (post hoc tests: *p* ≤ 0.04 for each). No differences were observed between >2.5–6 and >6–10 years post-IMRT, but lower mean FACT-Cog and NPCS scores were observed for >10 years compared to >2.5–6 years post-IMRT (post hoc: *p* < 0.05 for each).

**Conclusions:**

All QoL measures were low during the initial recovery period (≤2.5 years) and were higher by 6 years post-IMRT. At >10 years post-IMRT, lower scores were observed in the domains of NPC-specific and cognitive QoL. Survivors of NPC, even if treated with IMRT, are at risk for detriment in domain-specific QoL measures at very long-term follow-up.

## Background

Intensity-modulated radiation therapy (IMRT) is a major component of the standard treatment for nasopharyngeal carcinoma (NPC). Compared to conventional RT (CRT), IMRT provides superior conformity of the radiation dose to the tumor and greater sparing of adjacent organs, which in theory should translate to fewer adverse effects and better quality of life (QoL). However, there is evidence to suggest that IMRT, due to the low-dose “bath” effect, may still result in a risk of more subtle late normal tissue toxicities and could increase certain risks such as secondary malignancies compared to conventional RT [1–4].

From irradiation of the nasopharynx, even low doses of radiation to the proximate central nervous system could result in neurocognitive deficits, and it remains unclear to what extent IMRT reduces this effect compared to older methods. While studies have demonstrated neurocognitive deficits following CRT for NPC [5–10], there are almost no studies on neurocognitive deficits specifically following IMRT, especially over the very long term. One prospective study showed decreased neurocognitive function after IMRT, but only within a relatively short follow-up period of 12–26 months [11], when the combined profile of acute and late side effects could have still been evolving. Given that improved locoregional control from IMRT has now produced many NPC survivors who can live for decades following treatment, it is important to gain a better understanding of the long-term physical and cognitive effects of IMRT.

The aim of this study was to measure general, disease-specific, and cognitive QoL in NPC survivors treated with IMRT across a wide range of survival times. We hypothesized that variations in disease-specific and self-reported cognitive QoL domains would be observed among patients grouped by time since radiotherapy completion.

## Methods

This was a cross-sectional survey that included all surviving patients diagnosed and treated for NPC at our center with curative-intent IMRT, with or without chemotherapy. Only patients who were alive at the time of the study period between May and November 2013 were included. Any patient who finished treatment was included, and there was no minimum time required since completion of treatment to be eligible for this study. We excluded patients who had known recurrent disease, who received an RT modality other than IMRT (e.g. 3D conformal radiation therapy, or 3DCRT), and who were still undergoing active treatment.

All known surviving patients were contacted either by telephone or at the time of a routine follow-up appointment and offered participation over the study duration period between May and November 2013. At least three attempts were made to contact patients. If reached by phone, they were given the choice of completing the survey verbally by phone or having a written copy mailed to them with return postage included. Two validated questionnaires, FACT-NP and FACT-Cog, were completed once by each patient. For non-English speaking patients, translated written versions of the questionnaires were delivered and returned by mail. Institutional Review Board study pre-approval, informed consent processes, and secure data collection and storage procedures were observed during the conduct of the study.

FACT-NP is comprised of a general QoL measure (FACT-G) plus an NPC-specific measure (NPCS) that assesses site-specific symptoms such as dry mouth, hearing loss, etc. FACT-Cog was developed as a supplement to the FACT-G instrument, to measure health-related quality of life (HRQoL) symptoms specific to cognitive function. The hypothesis for this study was that there would be a significant difference among the cohorts in the more specific QoL domains which would be of greater magnitude than the general assessment (NPCS or FACT-Cog as opposed to FACT-G).

Scores for each questionnaire were computed following published guidelines obtainable from FACIT.org. Responses to each item in each questionnaire were scored on a scale of 0–4, with a higher number corresponding to better QoL. Items that reflected a negative symptom (i.e. “my mouth is dry”) were scored per the published guidelines, with the raw response inverted so that a higher number corresponds to better QoL. Patients were classified into 4 subsets by the time since they completed radiotherapy treatment (see below).

The FACT-NP questionnaire was scored out of 172. The NPC-specific subscale (NPCS) of FACT-NP was scored out of 64, and the FACT-G subscale of FACT-NP was scored out of 108 with 4 subscales which included functional well-being, emotional well-being, social well-being, and physical well-being. The FACT-Cog questionnaire was scored out of 132 with the summary score combining 4 QoL subscales, which included perceived cognitive abilities, perceived cognitive impairments, impact on quality of life, and comments from others. The published guidelines do not include thresholds regarding the interpretation of the scores.

One-way analysis of variance (ANOVA) methods were used to compare mean scores in the FACT-NP, FACT-G, NPCS, and FACT-Cog across the 4 cohorts of survivors. If overall differences were found with ANOVA methods (*p* < 0.05), then post-hoc pairwise comparisons using the Newman-Keuls test were performed to assess for differences between individual cohorts. Subscales were tested independently of their parent scores; for example, a significant difference in FACT-NP among groups was not a prerequisite to testing for differences in NPCS and FACT-G among groups.

As part of the ANOVA models, patterns of the mean scores were evaluated using linear contrast statements. One-way ANOVA was used to evaluate the effects of chemotherapy regimen on QoL and two-way ANOVA methods were used to evaluate effects of chemotherapy and time since IMRT. Fisher’s exact test was performed to compare proportions among the study cohorts. A probability value of less than 0.05 was considered to be significant. Statistical analyses were performed using STATA/SE, version 13. Except for post hoc tests no other adjustment for multiple comparisons was performed.

## Results

There were 243 patients identified in our records who were diagnosed and treated for NPC from 1995 to 2013. After applying exclusion criteria (RT other than IMRT, recurrent disease, deceased, active treatment), 160 patients were eligible for participation. Of these patients, 17 declined to participate, 20 had incorrect/outdated contact information, and 79 were unresponsive to repeated contact attempts. Ultimately, 44 patients agreed to participate in the study (44/160 = 27 % response proportion). After QoL data were collected, these patients were divided into four cohorts based on the duration since the end of radiotherapy. The median duration from treatment to completion of the questionnaires for the full study sample was 5.7 years (range: 5 months-16 years) with the interquartile range cutoffs at 2.5 and 10 years. To make the results more generalizable to other populations, the 4 subsets used for analysis were divided into ≤2.5, >2.5–6, >6–10 and >10 years. This resulted in reclassifying only 1 patient from the quartile distribution of our dataset. There were no other observations with values that fell between the quartile cutpoints and the endpoints that were used for analysis.

Table 1 shows the summary baseline characteristics of the patients who participated. The mean age at the time the questionnaires were completed was 55.5 years, and there was no significant difference in age among the cohorts (*p* = 0.20). Patients were predominantly Asian/Pacific Islander (API) and there were no differences in the proportion of API vs non-API patients among the cohorts. However, there was a significant difference in the proportion of non-English speakers among the cohorts (*p* = 0.005), due the predominance of non-English speakers in cohort 1 (≤2.5 years). In terms of disease stage (per AJCC 6th edition), there was no significant difference in the overall distribution among the four cohorts (I/II vs III/IV: *p* = 0.78). Even though there was no significant difference among the cohorts in terms of IMRT type, it is worth noting that cohort 4, those who were treated more than 10 years earlier, contained the only two patients in the study who were treated with the older technique of forward-planned IMRT rather than inverse-planned IMRT. Only 14 % of the patients were not treated with chemotherapy. Eighteen percent received concurrent chemotherapy and the majority, 68 %, received concurrent plus adjuvant chemotherapy. Among the four cohorts, there was a significant difference in terms of chemotherapy timing (none vs. concurrent vs. concurrent + adjuvant) (*p* = 0.04). However, there was no difference among cohorts as to the proportion treated with any chemotherapy (*p* = 0.26) (p-value not shown in Table 1).

**Table 1:** Summary Statistics of Nasopharyngeal Carcinoma Survivors Surveyed for Current Quality of Life (*n* = 44)

		Time since End of Radiotherapy Treatment				
Characteristic	Total (*n* = 44)	Cohort 1 ≤2.5 years (*n* = 11)	Cohort 2 >2.5–6 years (*n* = 13)	Cohort 3 >6–10 years (*n* = 9)	Cohort 4 >10 years (*n* = 11)	*p*-value
Mean time since end of RT (months)	74.6	15.6	49.0	93.6	144.3	
Range of time since end of RT (months)	5–162	5–28	31–68	69–116	128–162	
Age at time of treatment: mean ± SD (years)	49.2 ± 13.9	53.2 ± 9.5	52.8 ± 13.8	41.3 ± 14.6	48.6 ± 15.6	
Age at time of survey: mean ± SD (years)	55.5 ± 13.8	54.5 ± 9.7	57.3 ± 13.9	47.7 ± 14.7	60.6 ± 15.4	0.20
Gender						0.88
Men	35	9	10	8	8	
Women	9	2	3	1	3	
Ethnicity						0.13
Asian Pacific Islander (API)	34	11	8	7	8	
Non-API	10	0	5	2	3	
Language						**0.005**
English	31	3	10	8	10	
Non-English	13	8	3	1	1	
AJCC Stage (6th Ed.)						0.78
I or II	13	3	5	3	2	
III or IV	31	8	8	6	9	
Radiation Type						0.15
Forward-planned IMRT	2	0	0	0	2	
Inverse-planned IMRT	42	11	13	9	9	
Chemotherapy						**0.04**
None	6	1	4	0	1	
Concurrent Only	8	1	2	0	5	
Concurrent + Adjuvant	30	9	7	9	5	

A one-way analysis of variance was used to compare the age distributions among the cohorts and Fisher’s exact test was used for categorical variables. The probability values for statistically significant results (*p* < 0.05) are in bold

Significant overall differences in means due to the duration from treatment were observed for both FACT-NP and FACT-Cog scores (ANOVA: *p* = 0.002 and 0.02, respectively) (Table 2). The means for the two subscales of FACT-NP, both NPCS and FACT-G, each significantly differed among the 4 cohorts (*p* = 0.017, 0.002). The latter finding reflected the subdomains of FACT-G of functional well-being and emotional well-being which differed significantly among the 4 cohorts (*p* < 0.001, 0.003). Within FACT-Cog, the means for the subdomains of perceived cognitive abilities (PCA) and perceived cognitive impairments (PCI) were significantly different among the 4 cohorts (*p* = 0.01, 0.04). There was a significant quadratic effect for the overall pattern of scores for FACT-NP, FACT-G, NPCS and FACT-Cog measures (ANOVA quadratic contrast: *p* ≤ 0.004 for each). This reflected the higher QoL that was observed years >2.5–6 compared with the earliest cohort (≤2.5) which continued at years >6–10, followed by lower scores in each QoL measure after 10 years from IMRT. In terms of the subscales within FACT-G, a significant quadratic effect was observed for the domains of functional, emotional, and social well-being (*p* ≤ 0.05 for each), but not for physical well-being (*p* = 0.12). In terms of the subscales within FACT-Cog, a significant quadratic effect was observed for the domains of perceived cognitive ability, perceived cognitive impairment, and impact on QoL(*p* ≤ 0.05 for each), but not for comments from others (*p* = 0.07).

**Table 2:** Quality of Life Scores among Nasopharyngeal Carcinoma Survivors by Time since Radiotherapy (*n* = 44)

		Time since End of Radiotherapy Treatment Mean ± Standard deviation				
Item	Total (*n* = 44)	Cohort 1 ≤2.5 years (*n* = 11)	Cohort 2 >2.5–6 years (*n* = 13)	Cohort 3 >6–10 years (*n* = 9)	Cohort 4 >10 years (*n* = 11)	ANOVA *p*-value
FACT-NP Total (0–172)^a^	119.3 ± 24.7	98.8 ± 19.9	133.7 ± 18.9	128.5 ± 31.0	115.3 ± 14.4	**0.002**
NPC Subscore (NPCS) (0–64)	39.7 ± 11.1	34.9 ± 10.5	46.5 ± 9.2	41.9 ± 13.4	34.7 ± 7.5	**0.017**
FACT-G Component (0–108)	79.6 ± 17.0	63.9 ± 15.9	87.2 ± 11.1	86.6 ± 19.1	80.6 ± 12.7	**0.002**
Functional Well-being (0–28)	19.0 ± 6.0	13.1 ± 4.3	20.1 ± 4.6	22.3 ± 7.3	21.0 ± 3.6	**<0.001**
Emotional Well-being (0–24)	18.3 ± 4.2	14.5 ± 4.0	20.5 ± 2.5	19.3 ± 4.1	18.7 ± 4.2	**0.003**
Social Well-being (0–28)	21.2 ± 6.0	18.1 ± 6.4	23.8 ± 3.7	23.0 ± 5.5	19.9 ± 7.2	0.08
Physical Well-being (0–28)	21.0 ± 5.7	18.2 ± 7.1	22.8 ± 5.0	21.9 ± 5.9	21.0 ± 4.4	0.25
FACT-Cog Total (0–132)	98.9 ± 23.4	88.1 ± 22.9	113.2 ± 18.8	103.9 ± 20.9	88.9 ± 22.9	**0.02**
Perceived Cognitive Abilities (0–28)	19.5 ± 5.6	15.8 ± 5.3	23.0 ± 4.9	20.8 ± 6.2	18.2 ± 3.5	**0.01**
Perceived Cognitive Impairments (0–72)	54.6 ± 13.7	50.8 ± 14.1	62.3 ± 10.0	56.7 ± 10.4	47.8 ± 15.8	**0.04**
Impact on Quality of Life (0–16)	11.3 ± 4.5	9.0 ± 5.2	13.5 ± 3.9	12.2 ± 4.5	10.4 ± 3.2	0.07
Comments from Others (0–16)	13.4 ± 3.2	12.5 ± 3.9	14.4 ± 3.2	14.2 ± 1.6	12.5 ± 3.3	0.32

Results of one-way ANOVA for measuring overall differences in FACT-NP and its subscales, as well as FACT-Cog and its subscales, are shown among the cohorts. FACT-NP is comprised of the NPC-specific score (NPCS) plus the general QoL (FACT-G) score. FACT-G includes four subdomains that measure functional, emotional, social and physical well-being. FACT-Cog measures cognitive QoL and is comprised of four subdomains that measure perceived cognitive abilities, perceived cognitive impairments, impact on QoL and comments from others. Significant values (*P* < 0.05) are in bold

^a^Numbers in parenthesis indicate the range of possible scores

Post-hoc pairwise comparisons using the Newman-Keuls (NK) method were performed to help explain the overall significant differences in total scores and subscale scores among the cohorts (Tables 3 and 4). FACT-G was significantly lower for patients in cohort 1 (≤2.5 years post-IMRT) compared to each of the three other cohorts (*p* ≤ 0.01 for each). For FACT-NP, the overall difference resulted from the significantly lower mean score for cohort 1 (≤2.5 years) compared with cohorts 2 (>2.5–6 years) and 3 (>6–10 years) (post hoc tests: *p* = 0.003 and *p* = 0.01, respectively). For the NPCS component and for FACT-Cog the scores for cohort 2 (>2.5–6 years) were significantly higher compared with cohort 1 patients (≤2.5 years) and with cohort 4 (>10 years) (NPCS: *p* = 0.03 and *p* = 0.046; FACT-Cog: *p* = 0.04 and *p* = 0.03, respectively).

**Table 3:** *P*-values for Post Hoc Newman-Keuls Pairwise Comparisons (*n* = 44)

Comparison	FACT-NP	NPCS	FACT-G	FACT-Cog
Overall Probability from ANOVA	**0.002**	**0.017**	**0.002**	**0.02**
Pairwise comparisons by cohort:				
2 vs 1	**0.003**	**0.03**	**0.003**	**0.04**
3 vs 1	**0.01**	0.11	**0.002**	0.21
4 vs 1	0.08	0.96	**0.01**	0.93
3 vs 2	0.57	0.30	0.92	0.32
4 vs 2	0.12	**0.046**	0.55	**0.03**
4 vs 3	0.15	0.23	0.34	0.11

Significant *p*-values (*p* < 0.05) are in bold

**Table 4:** *P*-values for Post Hoc Newman-Keuls Pairwise Comparisons (Subscales) (*n* = 44)

Comparison	Functional Wellbeing (Subscale of FACT-G)	Emotional Wellbeing (Subscale of FACT-G)	Perceived Cognitive Abilities (Subscale of FACT-Cog)	Perceived Cognitive Impairments (Subscale of FACT-Cog)
Overall Probability from ANOVA	**<0.001**	**0.003**	**0.01**	**0.04**
Pairwise:				
2 vs 1	**0.002**	**0.003**	**0.01**	0.11
3 vs 1	**0.001**	**0.01**	0.07	0.29
4 vs 1	**0.002**	**0.01**	0.28	0.59
3 vs 2	0.57	0.48	0.30	0.32
4 vs 2	0.69	0.51	0.06	0.06
4 vs 3	0.54	0.70	0.25	0.25

Significant *p*-values (*p* < 0.05) are in bold

For the FACT-G subscales, both the functional well-being subscale and physical well-being subscale showed overall differences among the cohorts, and post-hoc tests showed that these subscales were significantly lower for patients in cohort 1 compared to each of the three other cohorts (*p* ≤ 0.01 for each). For the FACT-Cog subscales, the perceived cognitive abilities and perceived cognitive impairments subscales had overall significant differences among the cohorts. Post hoc testing showed that patients in cohort 1 had significantly lower scores for perceived cognitive ability (*p* = 0.01) compared to cohort 2. For perceived cognitive impairments, post-hoc testing failed to reveal any significant differences between the cohorts.

The lower NPCS and FACT-Cog scores for patients in cohort 4 (>10 years) did not appear to be due to older age or variation in cancer stage, as there was no statistically significant difference in these factors by cohort (Table 1). Furthermore, age was not correlated with FACT-NP, FACT-G, NPCS, or FACT-Cog scores (Pearson’s correlation: *p* = 0.90, 0.63, 0.64, 0.33, respectively). Cohort 4 included the only two patients in the study who received forward-planned IMRT, an inferior technique. The results of the analysis with these two patients excluded were identical to the full cohort (data not shown).

A one-way ANOVA was performed to investigate the effect of chemotherapy (none, concurrent only, concurrent plus adjuvant) on QoL measures and a significant difference was observed for only NPCS (*p* = 0.04) (Table 5). A post hoc analysis indicated that the difference in NPCS was due to lower scores in patients receiving concurrent chemotherapy alone or concurrent plus adjuvant chemotherapy compared to those who did not receive any chemotherapy (*p* = 0.03 and *p* = 0.02, respectively) (Table 6).

**Table 5:** Univariate Analysis of Quality of Life Scores by Extent of Chemotherapy: 1-Way Analysis of Variance (Mean ± Standard deviation) (*n* = 44)

Score	None	Concurrent Only	Concurrent + Adjuvant	*P*-value
Fact-NP	131.6 ± 20.5	122.7 ± 14.1	116.0 ± 27.2	0.34
NPCS	50.1 ± 6.9	37.3 ± 7.2	38.3 ± 11.6	**0.04**
Fact-G	81.6 ± 14.6	85.4 ± 12.2	77.6 ± 18.5	0.50
Fact-Cog	114.3 ± 14.5	96.0 ± 31.0	96.6 ± 22.0	0.23

Values are listed as mean ± standard deviation. Significant *p*-values (*p* < 0.05) are in bold

**Table 6:** Probability Values for Post Hoc Newman-Keuls Pairwise Comparison Tests of Nasopharyngeal Carcinoma Specific Scores (NPCS) by Chemotherapy Administration (*n* = 44)

Comparison	NPCS Probability Value
None vs Concurrent Only	***p*** **= 0.03**
Concurrent vs Concurrent + Adj	*p* = 0.83
None vs Concurrent + Adj	***p*** **= 0.02**

Significant *p*-values (*p* < 0.05) are in bold

Two-way ANOVA models were created for each QoL outcome with chemotherapy (none, concurrent, concurrent plus adjuvant) and time since IMRT completion (cohorts 1–4) as the two main effects. Testing for interaction effects was not performed due to small subset sample sizes and the distribution of variables. In these analyses, a significant difference for all 4 summary QoL measures was only due to the time since completion of IMRT (Table 7).

**Table 7:** Two-Way Analysis of Variance Model with Chemotherapy and Time since IMRT Completion as Main Effects (*n* = 44)

	Probability Values	
	Chemotherapy (3 groups)	Time since IMRT (4 groups)
FACT-NP	*p* = 0.40	***p*** **= 0.002**
NPCS	*p* = 0.11	***p*** **= 0.04**
FACT-G	*p* = 0.46	***p*** **= 0.002**
FACT-Cog	*p* = 0.43	***p*** **= 0.03**

For each QoL measure the main effects are: chemotherapy (none, concurrent only and concurrent plus adjuvant) and duration of time since IMRT as factor variables (cohorts 1–4). Significant *p*-values (*p* < 0.05) are in bold

## Discussion

To our knowledge this analysis presents the longest follow-up of post-IMRT quality of life that has been performed, and it is the first to demonstrate a temporal pattern in cognitive QoL in IMRT treated patients. Two key observations were made, the first being that the most recently treated patients (cohort 1, ≤2.5 years post-IMRT) had poorer general, cognitive and NPC-specific QoL compared to patients who were >2.5–6 years post-IMRT (cohort 2). This is consistent with prior studies in head and neck cancer patients demonstrating an initial drop in QoL following completion of cancer treatment [12–14]. The second and more compelling observation was that the cohort of patients who had the longest follow-up from treatment (cohort 4, >10 years) had poorer cognitive and NPC-specific QoL compared to recovered cohort 2 patients. While the difference between cohorts 1 and 2 might be explained by acute chemoradiation toxicity and initial recovery, the lower QoL for patients >10 years after radiotherapy raises the concern that IMRT, despite its technical sophistication, may still result in long-term deficits in site-specific and cognitive QoL many years after the recovery from acute toxicity.

The lower cognitive QoL in cohort 1 compared to cohort 2 is consistent with the study done by Hsiao et al. showing decreased cognitive function in the initial short-term period of 12–26 months after RT [11] and speaks to the effects of early chemoradiation toxicity. However, we were most interested in the distinctive findings manifesting in our long-term survivors. Our analysis of individual survey questions (data not shown) revealed that problems with verbal self-expression and short-term memory contributed to the difference in cognitive QoL scores between cohort 2 and cohort 4 patients. These symptoms are consistent with the possibility of subtle long-term radiation-induced damage to the temporal lobes, which frequently and uniquely occurs in the treatment of nasopharyngeal and other cancers of the skull base.

Aside from direct radiation damage to the brain parenchyma in the temporal lobes, radiotherapy of NPC confers a risk of long-term carotid artery damage [15], which could also result in cognitive impairment through the occurrence of cerebral ischemia. Radiation-induced hypothyroidism also commonly occurs in patients treated for head-and-neck cancer and could contribute to decreased attention and memory [16]. It would be useful if future studies could investigate the accumulated contributions of temporal lobe, carotid artery, and thyroid gland damage to overall radiation-induced cognitive decline.

Radiation toxicity is likely potentiated by the addition of concurrent and/or adjuvant chemotherapy, as we also noticed worsened NPCS scores in patients treated with both chemotherapy and radiation compared to radiation alone. This is consistent with other research that has demonstrated the increased acute and late toxicities of combined modality therapy [17]. Eventually, ongoing clinical trials aimed at further personalization of the de-intensification or intensification of systemic therapy administration may reduce the burden of long-term toxicities in select NPC patients [18].

Based on our results, it appears that cognitive decline might be most likely to be detected in surveys of patients approximately ten years after treatment. Thus, in the future clinical practice screening of cognitive function could be warranted at several years post-treatment to detect the onset of any neurocognitive deficits. With appropriate screening, it may be possible to address the worsening burden of late cognitive deficits by the use of pharmacologic or cognitive therapy, or in the preventive realm, to investigate amelioration of symptoms with neuroprotective agents in patients identified to be at high risk.

Radiation-induced brain injury has been theorized to occur through several potential mechanisms including depletion of vascular and glial clonogenic cells, depletion of neural stem cells, impaired synaptic plasticity, and inflammation [19]. Anti-inflammatory agents including PPAR-α agonists, PPAR-γ agonists, AT1 receptor antagonists, and renin-angiotensin system blockers have been under preclinical investigation as protectants against radiation-induced brain injury in mouse models [20–25]. A randomized controlled trial of memantine for the prevention of cognitive deficits due to whole brain radiotherapy found that patients treated with memantine experienced a significantly longer time to cognitive decline as measured by objective testing [26]. At this point, however, preventive strategies remain for the most part investigational.

On the other hand, the use of cognition-enhancing agents such as donepezil or modafinil has begun to enter practice. In a phase II single arm prospective trial, Shaw et al. demonstrated improved objective and self-reported cognitive function in 24 irradiated brain tumor patients after a 24-week course of donepezil [27]. This was followed by a recent randomized, placebo-controlled trial of 198 brain tumor patients which showed improvements in objective tests of cognitive function in the donepezil-treated patients relative to the placebo arm [28]. Modafinil has been shown to improve objectively-measured memory and attention skills in breast cancer survivors including those treated with radiotherapy [29], but the compound has not yet been investigated specifically for patients who experience radiation exposure directly to the brain. And finally, supportive non-pharmacologic approaches like cognitive-behavioral therapy, activity/exercise therapy, and aromatherapy have shown modest benefit in treating dementia [30] and might be of some therapeutic value in the context of radiation-associated cognitive decline.

The limitations of this study were 1) small sample size, 2) lack of baseline QoL measurements, 3) potential impact of technological evolutions, 4) potential participation bias, 5) reliance on self-reported rather than objective measurement of cognitive function and 6) potential confounding by age. With respect to the sample size, it prevented more extensive subset analyses. If the sample size were larger, we might have been able to test more nuanced hypotheses about the interrelated effects of factors such as age, disease stage, IMRT type, ethnicity, language, or gender. However, with only 44 patients participating in the study, it was infeasible to stratify by multiple variables.

With respect to lack of baseline QoL measurements, we recognize that not having these data as an internal control for each patient limited our ability to measure changes from baseline over time, particularly if there were unanticipated differences in medical comorbidities amongst the comparison groups. An excess of comorbid conditions might have been expected to have a time-dependent impact on physical and functional status on those individuals as the interval from treatment increased, although the ultimate effect on QoL is unknown. Although we believe that the observation of lower NPCS and FACT-Cog scores in our >10 year survivors compared to our 2.5–6 year survivors truly reflects a phenomenon of late radiation toxicity, it is not possible to guarantee that these two cohorts had similar baseline QoL, and the model was not able to account for potentially varying comorbidities across the cohorts. Obviously, a prospective cohort study with baseline data would be ideal.

With respect to the potential impact of technical evolution, it is possible that there was an effect of a “learning curve” with regards to radiotherapy technique, the precision of which may have gradually improved over the interval of time covered by this study. Poor precision of RT technique could feasibly result in greater acute and long-term toxicities. We believe the contribution of this effect in our study is minimal, but again, only a prospective cohort study controlling the radiotherapy techniques used would eliminate this possibility.

Additionally, the cross-sectional nature of this study meant that selection biases due to differential survival, follow-up losses or elective non-participation could not be controlled. By only including patients who were alive at the time of data collection and willing to participate, there was theoretically a selection bias in favor of higher QoL across all cohorts, but particularly in late survivors who may have outlived their treatment peers. Based on this we might have expected QoL to be artificially elevated in the late cohorts, but instead we observed that NPC-specific and cognitive QoL were lower only in the longest survivors. Thus it is unlikely that the findings in this study are simply a manifestation of selection bias.

Furthermore, we acknowledge the limitations of drawing conclusions about cognitive function based on self-reported QoL measures as opposed to objective testing. Indeed, the correlation between subjective and objective measures of cognitive function is not firmly established, and it remains unclear if differences in cognitive QoL are truly due to an independent effect or could simply be correlated to overall QoL. Objective testing of cognitive function could be done to address these concerns and corroborate subjective complaints in this domain. Nevertheless, for our intended purpose of characterizing subjective symptoms in patients across a wide range of survival times, we believe that these data remain useful in their own right.

Finally, while we did not find a correlation between age and FACT-NP or FACT-Cog scores within our study cohorts and did not detect a statistically significant difference in patient age among the four cohorts, the ability to detect these differences could have been limited by small sample size. Age may affect certain domains of QoL within the general population, especially in the realms of social/functional QoL and cognition. For example, in a study which measured FACT-G scores in 2,000 subjects (age ranging from 18 to 70+ years) from the general population in Austria, higher age was associated with lower QoL scores [31], and in post-hoc pairwise comparisons, subjects who were 70+ years in age had somewhat lower FACT-G scores compared to other age groups. On the other hand, in an Australian study measuring FACT-G in 9,419 subjects (age ranging from 20 to 75 years) from the general population, there was no correlation between age and FACT-G scores [32]. FACT-NP has not been evaluated in a general population but FACT-Cog has been. In a French study, FACT-Cog scores were measured among 213 healthy subjects ages 35–89 and it was found that for the PCI (perceived cognitive impairment) and PCA (perceived cognitive abilities) subscales, subjects aged 70–89 had significantly lower scores compared to all other age groups [33].

However, it does not seem that our findings are not entirely explained by an effect of age. Although age is likely related to certain domains of QoL, it is unclear if this is a linear correlation or more likely, if there is a threshold above which a decline occurs. To wit, the prior QoL studies detected statistically significant difference in subjects age 70 and above, while all other age groups were not different from each other. In our study, the mean age of our oldest age group (cohort 4, *n* = 11) was 60.6 years and within this cohort, only three patients were aged 70 or above. In addition, we noticed that cohort 2 of our study had absolute FACT-Cog scores that were similar to population norms published in the French study, but cohort 4 of our study appeared to have absolute FACT-Cog scores that were actually lower compared to the corresponding population norms, even compared to the oldest age group (70–89 years) in the French study. Thus, while it is possible that age accounted for some of the change in QoL observed in this study, it is unlikely to explain the entire effect.

In the future, a prospective cohort study would be ideal to confirm the relationship between IMRT and the QoL changes over the long term, but the cost of this strategy over the period of 15+ years of follow-up needed to see late-term cognitive changes may be prohibitive. The results presented herein already yield important insights into the late deficits that occur in IMRT-treated patients and provide clinically relevant data useful to providers in counseling NPC patients about the expected quality of life associated with long-term survivorship. These findings may stimulate the further development of approaches to characterize and manage the long-term adverse effects of nasopharyngeal cancer treatment.

## Conclusions

In patients treated with IMRT for nasopharyngeal cancer, quality of life measures are lower during the initial recovery period (≤2.5 years) and were higher by 6 years post-IMRT. At >10 years post-IMRT, lower scores are observed in the domains of NPC-specific and cognitive QoL. Despite improved survival and toxicity outcomes in the era of IMRT, survivors display nasopharyngeal-specific and cognitively related quality of life decreases at the very long term.

## Abbreviations

3DCRT: Three-dimensional conformal radiation therapy
AJCC: American Joint Committee on Cancer
ANOVA: Analysis of variance
API: Asian/Pacific Islander
CRT: Conformal radiation therapy
FACT: Functional assessment of cancer therapy
FACT-Cog: Functional assessment of cancer therapy - cognitive
FACT-G: Functional assessment of cancer therapy - general
HRQoL: Health-related quality of life
IMRT: Intensity-modulated radiation therapy
NK: Newman-Keuls
NPC: Nasopharyngeal carcinoma
NPCS: Nasopharyngeal carcinoma-specific
PCA: Perceived cognitive abilities
PCI: Perceived cognitive impairments
QoL: Quality of life
RT: Radiation therapy
